# Cost-Effectiveness of MRI-Based Identification of Presymptomatic Autism in a High-Risk Population

**DOI:** 10.3389/fpsyt.2020.00060

**Published:** 2020-02-19

**Authors:** Ian O. Williamson, Jed T. Elison, Jason J. Wolff, Carlisle Ford Runge

**Affiliations:** ^1^ School of Public Health, University of Minnesota, Minneapolis, MN, United States; ^2^ Institute of Child Development, University of Minnesota, Minneapolis, MN, United States; ^3^ Department of Pediatrics, University of Minnesota, Minneapolis, MN, United States; ^4^ Department of Educational Psychology, University of Minnesota, Minneapolis, MN, United States; ^5^ Department of Applied Economics, University of Minnesota, Saint Paul, MN, United States

**Keywords:** autism spectrum disorder, magnetic resonance imaging, cost-effectiveness, screening, early intensive behavioral intervention

## Abstract

Biological siblings of children with autism spectrum disorder (ASD) have increased risk of receiving an ASD diagnosis. In the U.S., most children with ASD are diagnosed after the optimal age to initiate early intervention which can reduce symptom severity and improve outcomes. Recent evidence suggests magnetic resonance imaging (MRI) in the first year of life can predict later diagnostic status in high-risk siblings. We investigated whether MRI-based screening is a cost-effective method for assigning early intervention. A hybrid decision tree/Markov model was used to evaluate two MRI-based screening strategies at 6 and 12 months of age. Primary outcomes were costs in U.S. dollars and quality-adjusted life years (QALYs). Results were reported as incremental cost-effectiveness ratios (ICERs). Costs were estimated from societal, health care, and educational perspectives. One-way and probabilistic sensitivity analyses were performed. From a societal perspective, the ICER for MRI-based screening at 6 months was $49,000 per QALY when compared to the status quo, implying that such screening is cost-effective at willingness-to-pay (WTP) thresholds of $50,000–$100,000 per QALY. From the health care and educational perspectives, the ICERs were larger at $99,000 and $76,000 per QALY, respectively. Sensitivity analysis identified that the parameters most influential in affecting cost-effectiveness were the prevalence of ASD and/or co-occurring intellectual disability. MRI specificity also has significant impacts which add to the uncertainty of the results. Future work is needed to determine the sensitivity and, in particular, the specificity of MRI with more certainty. Notably, the cost of the MRI-based screening had the least impact.

## Introduction

Autism spectrum disorder (ASD) is a neurodevelopmental disorder characterized by impaired social–communication skills and the presence of restricted and repetitive behavior. Symptoms vary in range and severity, imposing burdens which are deeply personal for families and posing numerous societal, health care, and educational challenges. Accumulating evidence indicates that early intervention is measurably helpful in meeting these challenges. This study quantifies these effects by examining the cost-effectiveness of implementing magnetic resonance imaging (MRI) in the first year of life to augment early identification of risk and accelerate entry into intensive behavioral interventions.

Prevalence estimates of ASD in the United States range from one in 59 in the general population to one in five for children with older siblings diagnosed with ASD. (1–3) The lifetime cost, including housing, special education, and productivity loss, of supporting an individual with ASD, compared to those without it, is estimated at between $1.4 and $2.4 million. (4) Total costs in the U.S. may exceed $1 trillion annually by 2025. (5) Reliable early diagnoses of ASD can be made between 18 and 36 months of age. (6) Early intensive behavioral intervention (EIBI), assumed to be initiated before age 4—a time of rapid development and substantial brain plasticity—can produce significant long-term gains in social, cognitive, and language development. (7–9) Although ASD can be reliably diagnosed earlier, the median age of diagnosis in the U.S. is 50 months of age. (1, 6) As a consequence, more than half of U.S. children with the disorder are diagnosed after age 4, too late to receive the full benefits of early intervention, while even those diagnosed prior to age 4 often experience significant delays in treatment. (10, 11) Improving early identification of ASD therefore increases opportunities for early intervention.

Two reports, using MRI at 6 and 12 months of age, predict with greater than 80% accuracy whether high-risk infant siblings of children with autism will subsequently meet diagnostic criteria for ASD at age 24 months. (12, 13) These findings raise the possibility of prodromal or presymptomatic identification of ASD among children at high risk and prompt an essential public health question: Is it cost-effective to identify and assign treatment based on early MRI screening?

A recent study found that reducing wait times in Canada for children with ASD to receive behavioral intervention was cost-effective and, in fact, cost saving. (14) Our objective was to determine if using MRI to assign early intervention could produce similar results in a U.S. setting due to its ability to identify ASD in children as early as 6 months of age—at least 1 year earlier than other available screening tools. We performed a cost-effectiveness analysis of assigning EIBI based on early MRI identification methods in infants at elevated risk for ASD from three perspectives. The first and second are from the health care system and educational perspectives and the third from the perspective of society as a whole. (15) The health care and educational perspectives only consider costs borne directly by their sector, such as medical care or special education. The societal perspective is a wider view, capturing costs related to housing, caregiver time, and indirect costs such as foregone caregiver productivity. The assumed objective was to maximize life expectancy and quality of life (QOL) for children diagnosed with ASD while considering the varying ASD-related costs of each perspective.

## Materials and Methods

### Study Population

The target population in our analysis is a U.S. birth cohort of high-risk infant siblings who have an older biological sibling already diagnosed with ASD. (2, 3) A child with ASD may or may not also have an intellectual disability (ID), defined as an intelligence quotient below 70, coupled with functional impairment. Individuals with both autism and ID have worse lifetime outcomes than those without ID for life expectancy, QOL, and associated costs. (4, 16, 17) We assumed ASD is a lifelong disability and childhood-onset ID persists into adulthood.

### Decision Analytic Model

Our decision analytic model uses a combination of decision tree and Markov model. Decision analysis considers decision-making under uncertainty and can be used to evaluate health care interventions such as MRI-based screening for autism. It is organized around “decision trees” that involve choices (decision nodes) and random events (chance nodes). By “folding back” the decision tree sequences, the most cost-effective outcome can be isolated. (18) To evaluate an MRI-based approach as a potential risk classification tool, we considered four alternative decision strategies: (1) Status Quo, (2) Test & Treat–6 months, (3) Test & Treat–12 months, and (4) Treat All. Strategy (1) is the reference case for our comparisons. In this strategy, some, but not all, children diagnosed with autism may develop symptoms leading to ASD identification before age 4 and thereby receive early intervention. Consequently, a certain percentage of children do not receive intervention early enough to avoid co-occurring ID. This reference case represents the status quo in the U.S.

**Figure 1:**
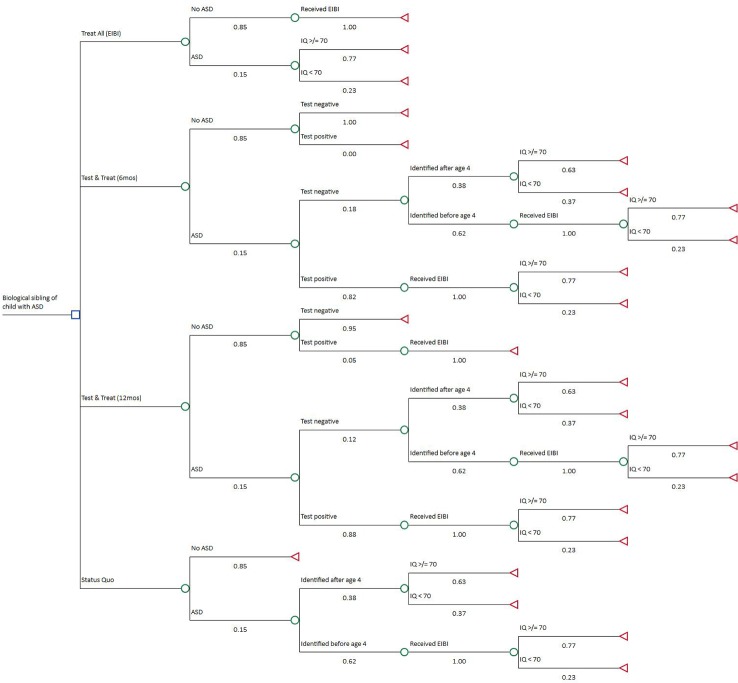
Decision Tree. The decision node includes four strategies from top to bottom: Treat All, Test & Treat–6 months, Test & Treat–12 months, and Status Quo. Expected lifetime costs and quality-adjusted life years (QALYs) were calculated at each terminal node using a two-state live or die Markov model.

A Treat All (i.e., high-risk siblings) strategy (4) is included because a risk classification tool is only useful if it provides information that might change a decision to treat or not. The Treat All strategy assumes that all high-risk children receive early intervention, as illustrated by a recent randomized intervention trial conducted in high-risk infant siblings of children with autism, (19) initiated before age 4 regardless of ASD symptoms. Under this strategy, many children would receive unnecessary and resource-intensive intervention. Each test strategy (2) and (3) involves performing one of the two MRI screening methods on each child in the first year of life and providing early intervention to those who screen positive. Negative findings continue to be monitored as if they were in the Status Quo strategy; some may be identified as false negatives in time to receive early intervention as well. False positives receive treatment and, just as in the Treat All strategy, do not incur any negative effect from early intervention other than cost. We calculate expected lifetime outcomes at the end nodes of each strategy for a cohort of 6-year-old children. TreeAge Pro 2018 software was used to perform the analysis (TreeAge Software, Inc.). **Figure 1** is a diagram of the decision tree.

Parameter estimates and ranges tested during sensitivity analysis for all branch probabilities and outcomes in the decision tree are shown in **Table 1**, including the model parameters, the base case, range, and data sources. The prevalence of ASD within the cohort is the same for each alternative. There are two possible outcomes for a child with ASD: a lifetime with ASD accompanied by no ID or a lifetime with ASD and co-occurring ID. A key to our analysis is that the probability of ID varies across the model’s alternatives depending on whether behavioral intervention is initiated by age 4. A primary effect of early intervention is increased IQ, which results in a lower risk ratio for co-occurring ID. (7) The risk ratio was calculated by adding the IQ gain to a base IQ distribution of children with ASD and comparing the new and base proportions of those with IQ < 70. Early screening in each of the test strategies increases the likelihood of receiving early intervention even if no symptoms are present by age 4. Payoffs for the decision tree were calculated using a Markov model. Discounted lifetime ASD-related costs and quality-adjusted life years (QALYs) were estimated from age 6 until calculated life expectancy using a “live or die” Markov model. Mortality during each cycle was based on the 2012 U.S. Life Tables plus an ASD-related increased risk. (24) Individuals with ASD have a two to three times greater mortality rate than the general population, with co-occurring ID associated with even greater risk. (16, 25)

**Table 1:** Model parameters.

Parameter	Mean estimate	Range tested	Probabilistic sensitivity analysis		Source
			Standard deviation	Distribution		
Prevalence of ASD among biological siblings	0.15	0.01–0.19	0.01	Beta	(2)
Sensitivity of MRI screening–6 months	0.82	0.48–0.97	n/a	n/a	(12, 13)
Sensitivity of MRI screening–12 months	0.88				
Specificity of MRI screening–6 months	1	0.90–1.00	n/a	n/a	
Specificity of MRI screening–12 months	0.95				
Probability of receiving early intervention	0.62	0.48–0.76	0.07	Beta	(1)
Prevalence of ID without early intervention	0.37	0.10–0.63	0.04	Beta	(20)
Risk ratio of ID with early intervention	0.61	0.32–1.00	0.20	Beta	(7)
Mortality hazard ratio (with ASD)	2.18	2.00–2.38	0.10	Gamma	(16)
Mortality hazard ratio (with ASD and ID)	5.78	4.94–6.95	0.46	Gamma	
QOL weight (with ASD)	0.68	0.47–0.70	0.23	Beta	(17, 21–23)
QOL weight (with ASD and ID)	0.46	0.34–0.58	0.17	Beta	
Cost of MRI screening	$1,814	$1,288–$2,464	n/a	n/a	HCCI
Cost of early intervention	$148,367	$112,958–$183,777			(4)
Annual costs (without ASD)	$0	$0			Assumption
Annual costs (with ASD)					
Ages 6–17	$58,526	± 25%	n/a	n/a	(4)
Ages 18–21	$69,889				
Ages 22+	$54,154				
Annual costs (with ASD and ID)					
Ages 6–17	$95,983	± 25%	n/a	n/a	(4)
Ages 18–21	$127,718				
Ages 22+	$96,247				
Discount rate	3%	1%–5%			Assumption

ASD, autism spectrum disorder; ID, intellectual disability; QOL, quality of life; n/a, not available.

The effects of variability in key parameters were tested using one-way sensitivity analysis. Minimum and maximum values were selected from the reported ranges and confidence intervals related to the point estimates. Joint parameter uncertainty was tested with a probabilistic sensitivity analysis (PSA).

### Costs

Cost estimates are based on a recent analysis presenting the difference in lifetime autism costs with and without ID. (4) This analysis found differences between the two outcomes for medical, educational, residential accommodations, and other non-medical cost categories. For other categories, such as lost productivity for individuals with ASD, there were no differences between those with or without ID. We nonetheless included such costs to reflect the difference in life expectancies associated with ASD and ID comorbidity. This is a conservative approach because excluding them would bias the model in favor of test and treatment strategies by not accounting for additional costs from gained life years. (20)

The costs of early intervention include 2 years of ASD-related special education. The societal perspective also includes 2 years of non-medical costs and parents’ productivity losses. MRI cost is a national average for brain MRI scans taken from a website created by the Health Care Cost Institute. All costs are inflated to 2018 U.S. dollars using the Personal Consumption Expenditures–Healthcare Index for medical costs and the Consumer Price Index for all other costs. (15) An impact summary, included in the **Supplementary Material**, breaks down which costs were used for the societal, health care, and educational perspectives.

### Quality-Adjusted Life Years

The QALY is a measure of both length and quality of life. It is a recommended measure because it allows for comparisons with other diseases, interventions, and/or policies. (15) Estimates for the terminal node Markov models’ QOL weights were derived using a weighted average of several reported Health Utilities Index III (HUI3) scores for children with any type of autism diagnosis. (21–23) In order to estimate the QOL weights for our two ASD-related outcomes, a negative QOL adjustment was applied for ID. (17) Specifically, we assumed a comorbid ID prevalence of 0.32 and calculated the resulting QOL weights for children with ASD only and children with ASD and ID. QOL weights were defined as 1 for individuals without ASD and 0 for death. While QOL weights of 1 and 0 lifetime costs for the non-ASD population are not accurate assumptions, the model’s results are not affected. The difference in costs and QALYs between each strategy is 0 because the number of individuals without ASD does not change across any strategy. QOL weights were assumed to be stable during childhood and decreasing with age in adulthood. (26) In addition to the QALY, relative risk of ID comorbidity was measured as an intermediate outcome.

### Cost-Effectiveness

Cost-effectiveness results are presented as incremental cost-effectiveness ratios (ICERs). The numerator of the ICER is the difference in lifetime costs between two comparators, and the denominator is the difference in lifetime QALYs. This allows for an ordering of increased effectiveness of treatments compared to competing alternatives. Any strategy that is more effective and less expensive than the previous one will “dominate” the other. A strategy that is more effective and more expensive may be considered cost-effective if the ICER is below the willingness-to-pay (WTP) threshold assumed. In other words, depending on society’s willingness to bear additional costs, more effective treatment strategies may be considered. The most cost-effective strategy is the one with the greatest ICER that is still below the WTP threshold. We assumed the two most commonly used U.S. WTP thresholds of $50,000 and $100,000 per QALY. (27) We also applied a 3% discount rate to both costs and QALYs.

## Results

### Base Case

**Table 2** presents the results from our base case analysis of the four strategies and three perspectives in terms of lifetime costs and cost-effectiveness measures. There was no dominant strategy, which means that none was simultaneously more effective and less expensive than any other. ICERs are calculated from least to most effective strategy. Status Quo was the least effective strategy. From the societal perspective, the test strategy with 6-month MRI resulted in an ICER of $49,000/QALY, and ICERs for the other strategies exceed a million dollars indicating only slightly more effectiveness gained at significantly higher costs. The results are less dramatic, but similar, when referencing the Status Quo strategy as a common baseline instead of comparing strategies incrementally. The ICER for the 12-month Test & Treat strategy is $169,000 per QALY, and the Treat All ICER is greater than $2 million per QALY.

**Table 2:** Cost-effectiveness results.

Strategy	Lifetime Costs	QALYs	ICER	ICER (ref. common baseline)	ID (prevalence)	ID (relative risk)
**Societal Perspective**
Status Quo	$294,586	27.0084	n/a	n/a	0.0421	1
Test & Treat (6-month)	$296,949	27.0563	$49,331	$49,331	0.0353	0.8397
Test & Treat (12-month)	$303,294	27.0598	$1,810,850	$169,435	0.0348	0.8280
Treat All	$421,367	27.0668	$16,846,890	$2,170,729	0.0339	0.8045
**Health Care Perspective**
Status Quo	$78,170	27.0084	n/a	n/a	0.0421	1
Test & Treat (6-month)	$82,929	27.0563	$99,369	$99,369	0.0353	0.8397
Test & Treat (12-month)	$87,659	27.0598	$1,349,813	$184,626	0.0348	0.8280
Treat All	$172.054	27.0668	$12,041,723	$1,607,478	0.0339	0.8045
**Educational Perspective**
Status Quo	$47,303	27.0084	n/a	n/a	0.0421	1
Test & Treat (6-month)	$50,963	27.0563	$76,417	$76,417	0.0353	0.8397
Test & Treat (12-month)	$55,745	27.0598	$1,364,739	$164,257	0.0348	0.8280
Treat All	$142,059	27.0668	$12,315,474	$1,622,403	0.0339	0.8045

ICER, incremental cost-effectiveness ratio; ID, intellectual disability; QALY, quality-adjusted life year; n/a, not applicable.

Lifetime costs from the health care and educational perspectives were significantly lower for all strategies. However, the ICERs follow a similar pattern to those in the societal perspective. ICERs for the health care and educational perspectives are below $100,000 per QALY for the 6-month Test & Treat strategies ($99,000 and $76,000, respectively) then exceed $1 million per QALY for the 12-month Test & Treat strategies. In terms of the intermediate outcome, ID comorbidity, the relative risk of ID when compared to the Status Quo reference case ranged from 0.80 to 0.84 for the three other strategies.

### Sensitivity Analysis

**Figure 2:**
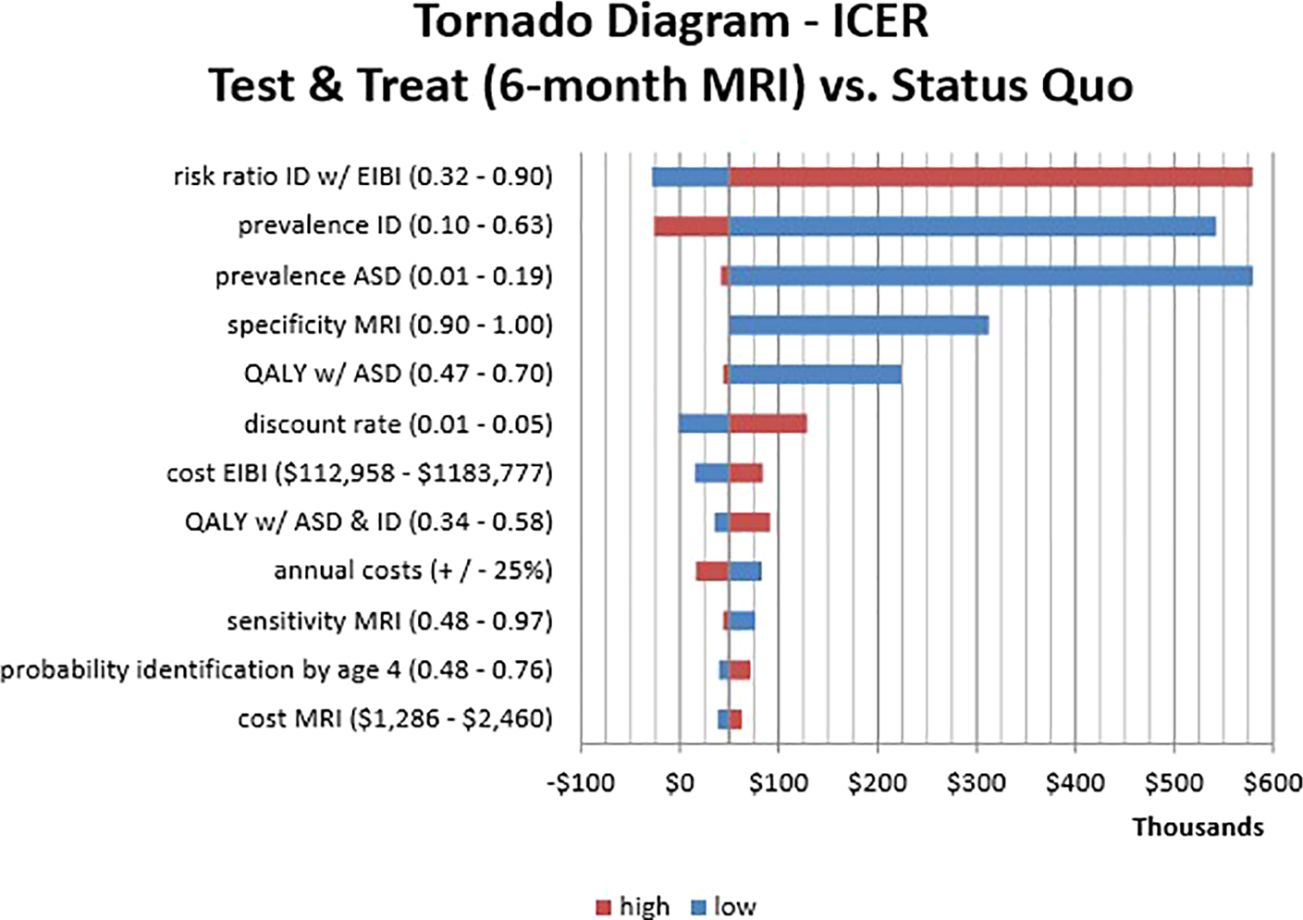
One-Way Sensitivity Analysis Tornado Diagram. This visualization identifies which parameters affect the incremental cost-effectiveness ratio (ICER) of Test & Treat strategy (6-month MRI) compared to the Status Quo. The horizontal axis shows ICER values while the vertical axis shows parameter estimates with corresponding high/low input values. The bar length is the change in ICER that results from varying the parameters across the specified range. Bar colors indicate the direction of the input value (high/low) and the resulting ICER.

Results of the one-way sensitivity analysis are presented only for the societal perspective as a “tornado diagram” in **Figure 2**. The diagram shows the effect of varying each parameter input in determining the ICER for the 6-month Test versus Status Quo strategies. The bar width indicates the range of calculated ICERs across the range of values. The greatest sensitivity was from the risk ratio of ID with early intervention. This was expected because the key component in our analysis was varying the probability of ID across strategies depending on the provision of behavioral intervention before age 4. Our model was also sensitive to our estimates of the prevalence of ID and ASD. By contrast, varying the intervention and screening costs did not have a significant impact on the ICERs, likely due to the greater influence of lifetime costs, which were somewhat sensitive to the discount rate. For the diagnostic parameters of the MRI screening methods, it was clear that test specificity is a key driver of the results. As can be seen in the tornado diagram, even slight reductions in specificity raise the ICER above the WTP threshold. Specificity had a much greater impact on our results than test sensitivity likely due to the high intervention costs resulting from false positives.

PSA results are presented in **Supplementary Figure 1** as an incremental cost-effectiveness scatter plot comparing the Test & Treat (6 months) strategy with the Status Quo strategy. Over half (52%) of the 10,000 Monte Carlo simulations resulted in a QALY gain for the Test & Treat (6 months) strategy with an ICER less than $100,000/QALY and 41% were less than $50,000/QALY. Twenty percent of simulations were superior with positive incremental effectiveness and negative incremental costs, i.e., cost savings.

## Discussion

### Interpretation of Results

We performed a cost-effectiveness analysis to determine whether MRI-based screening of high-risk infant siblings of children with autism represents a cost-effective solution to augment early identification and increase the likelihood of lower age of enrollment in early intervention. It bears emphasis that despite the economic and social costs of ASD, there have been few economic evaluations related to early intervention. (28) Prior cost–benefit analyses found that high initial costs associated with intensive behavioral intervention were overtaken by the positive impacts of significant long-term savings. (29, 30) A cost-effectiveness analysis of reducing wait times between diagnosis and treatment in Canada found substantial cost savings, in addition to significant gains in adult outcomes. (14) Only one economic evaluation was identified that used the QALY as a measure of effectiveness as recommended by the Second Panel on Cost-Effectiveness in Health and Medicine.(15) However, the QALY estimates in that study were based solely on employment status, not autism.(31)

Our analysis reveals that MRI-based screening of biological siblings may be a cost-effective method for assigning behavioral treatment because the ICERs fall within commonly used WTP thresholds of $50,000 and $100,000 per QALY. (27) Providing early intervention to everyone would be more effective but significantly exceed any commonly accepted WTP thresholds, even in a high-risk population such as younger biological siblings of children with ASD. Using MRI-based screening to assign early intervention could realize most of the same benefits as providing the behavioral treatment to everyone in a high-risk population, at costs nearly $100,000 less per child. The cost of an MRI is roughly 1/80 that of intensive behavioral intervention. Our one-way sensitivity analysis suggested the cost of the MRI would have the least impact on the results of our cost-effectiveness analysis. This was consistent for all three perspectives (health care, societal, and educational).

The fact that lower lifetime costs from the health care and educational perspectives did not translate into lower ICERs is likely due to the high upfront costs of early intervention assumed to be relevant from both perspectives. Not only are the costs of early behavioral intervention significant, they must be paid up front, while benefits are accrued over a lifetime. (32) The negative long-term consequences of late ASD identification, preventing early intervention, are the most significant costs to society and also affect the health care and educational systems. In our analysis of alternative perspectives, we assumed 100% of the direct costs of behavioral treatment were borne by each of the health care and educational systems. These significant up-front costs are likely to be shared; therefore, MRI-based assignment to early intervention is even more likely to be cost-effective from both perspectives. However, even if it were to be cost-effective, it would not guarantee accessibility to MRI screening for all children and cannot answer the question of affordability without performing a budget impact analysis. While our target population was biological siblings of children with ASD, these results may be of relevance to other populations at elevated risk for ASD or other psychiatric or neurodevelopmental disorders.

### Limitations

This analysis has several limitations. First, we did not account for symptom severity other than IQ. This oversimplifies the multifaceted nature of ASD. For example, the QOL weights we used do not accurately reflect the possibility of an individual with ASD and ID achieving independence and having a better QOL compared to an individual with ASD and high IQ who presents debilitating symptomatology and is completely dependent. Including other consequences of ASD in the model could have a major impact on the results. Second, our QALY estimates were limited by the small number of ASD-related QOL studies available to derive them. For reasons described by Grosse et al. (33), deriving QALY estimates for ASD and/or ID is challenging, especially among children. (33) Moreover, we assumed that QOL differences based on ASD and ID would remain stable into adulthood, although the estimates were based only on children. If the QOL difference actually narrows with age, then our results would overstate the effectiveness. However, the QALY is a recommended effectiveness measure for economic evaluations and therefore used in our analysis. (15) To account for this greater uncertainty in the QALY parameters, we allowed for wide overlapping ranges in sensitivity analyses. This led to some negative incremental QALYs in our PSA even though we assumed no negative effect from early intervention. Third, in addition to the QALY estimates, there is substantial uncertainty around intervention effectiveness and costs which, if our assumptions are wrong, would significantly alter our model results and conclusions. For example, other recently published estimates of ASD-related costs are substantially lower than those of Buescher et al. but do not distinguish costs based on presence of ID (4, 34). Using the higher annual cost estimates in our model risks overstating the lifetime costs of ASD, thereby overstating the benefits of early intervention and MRI screening. Fourth, the assumption that false positives and early intervention have no negative effect other than cost is unlikely. It may be true for children in their toddler years but does not include the negative QOL impacts on a child’s caregiver(s), for example.

Finally, model parameters used for the MRI characteristics were based on only two studies. Specificity approaching 1.00 is likely unrealistic, and adjusting this parameter downward even modestly was associated with substantially higher ICERs. For example, using a specificity of 0.98 increases the ICER to $102,000 for the societal perspective, thereby exceeding the WTP thresholds. In addition, we cannot be sure that the differences in sensitivity and specificity between the 6-month and 12-month MRIs represent true differences in diagnostic parameters at the two time points. It could be due to sensitivity of the sequences (i.e., fcMRI or sMRI), over-optimistic specificity at 6 months, or developmental change between 6 and 12 months of age. The 12-month MRI used deep learning and the 6-month MRI used a basic support vector machine which are slightly different machine learning techniques. For this reason, we chose not to combine the studies into one hypothetical MRI with a sensitivity/specificity distribution for the PSA. It is possible the difference between time points is simply random variance around the true sensitivity and specificity of the test meaning the decision of when to use the MRI, at 6 or 12 months of age, is irrelevant. This is a limitation of the analysis. Future work is necessary to better define model parameters. Subsequent efforts may consider MRI as a level 2 screener in order to optimize specificity estimates. These studies also used research-quality MRI scans which may not perform the same as standard clinical MRIs. The feasibility of using the MRI outside a research study remains unknown and, therefore, coupled with the impact of prevalence assumptions in our model, these results are not generalizable to larger settings or the overall population.

### Conclusions

MRI-based screening may be a cost-effective method for assigning treatment to children at higher risk of ASD. However, due to the small set of MRI studies used to parameterize the model, there is much uncertainty surrounding this finding, especially from the health care or educational perspective. MRI-based universal screening in a low-risk population would likely provide marginal improvements at excessive cost. This does not negate the potential utility of MRI-based approaches to complement traditional universal screening approaches if implemented as the second or third stage of a screening sequence. To enhance future cost-effectiveness analyses, further research into the MRI test characteristics and QOL scores related to autism is needed, particularly measuring the preference-based QOL effects of early intensive behavioral intervention.

## Data Availability Statement

The datasets generated for this study are available on request to the corresponding author.

## Author Contributions

IW and CR developed the conceptual idea. IW designed the model, performed the calculations, and wrote the initial draft. JE and JW verified the parameters and supervised the analysis. All authors discussed the findings and contributed to the final manuscript.

## Funding

IW was supported by UMN National Research Service Award in Health Services Research Project Number T32 HS 00036. JE was supported by R01 MH104324 and JW was supported by K01 MH101653, both from the National Institutes for Mental Health (NIMH).

## Conflict of Interest

The authors declare that the research was conducted in the absence of any commercial or financial relationships that could be construed as a potential conflict of interest.
