# Supplementary material for: Cost-Effectiveness of MRI-Based Identification of Presymptomatic Autism in a High-Risk Population
Source: Front Psychiatry. 2020 Feb 19;11:60. doi: 10.3389/fpsyt.2020.00060 (PMC7042195; doi:10.3389/fpsyt.2020.00060)

Figure S1. Incremental Cost-Effectiveness Scatter Plot.

This figure shows the “cloud of uncertainty” of a cost-effectiveness plot comparing the Test & Treat (6 months) strategy with the Status Quo strategy. The horizontal axis depicts the gain in QALYs; the vertical axis depicts the incremental cost in dollars, the two components of the ICER: the first the denominator and the second the numerator. Each blue dot represents the incremental cost-effectiveness of one Monte Carlo simulation (out of 10,000). Blue dots appearing in the right half of the plot represent simulations with gains in QALYs and those appearing in the lower right quadrant represent simulations with an ICER less than zero (i.e. cost-saving). The two dashed lines depict the WTP thresholds of $50,000 and $100,000 per QALY. Dots to the right and below of the dashed line have ICERs less than the respective threshold indicating an iteration that was cost-effective.

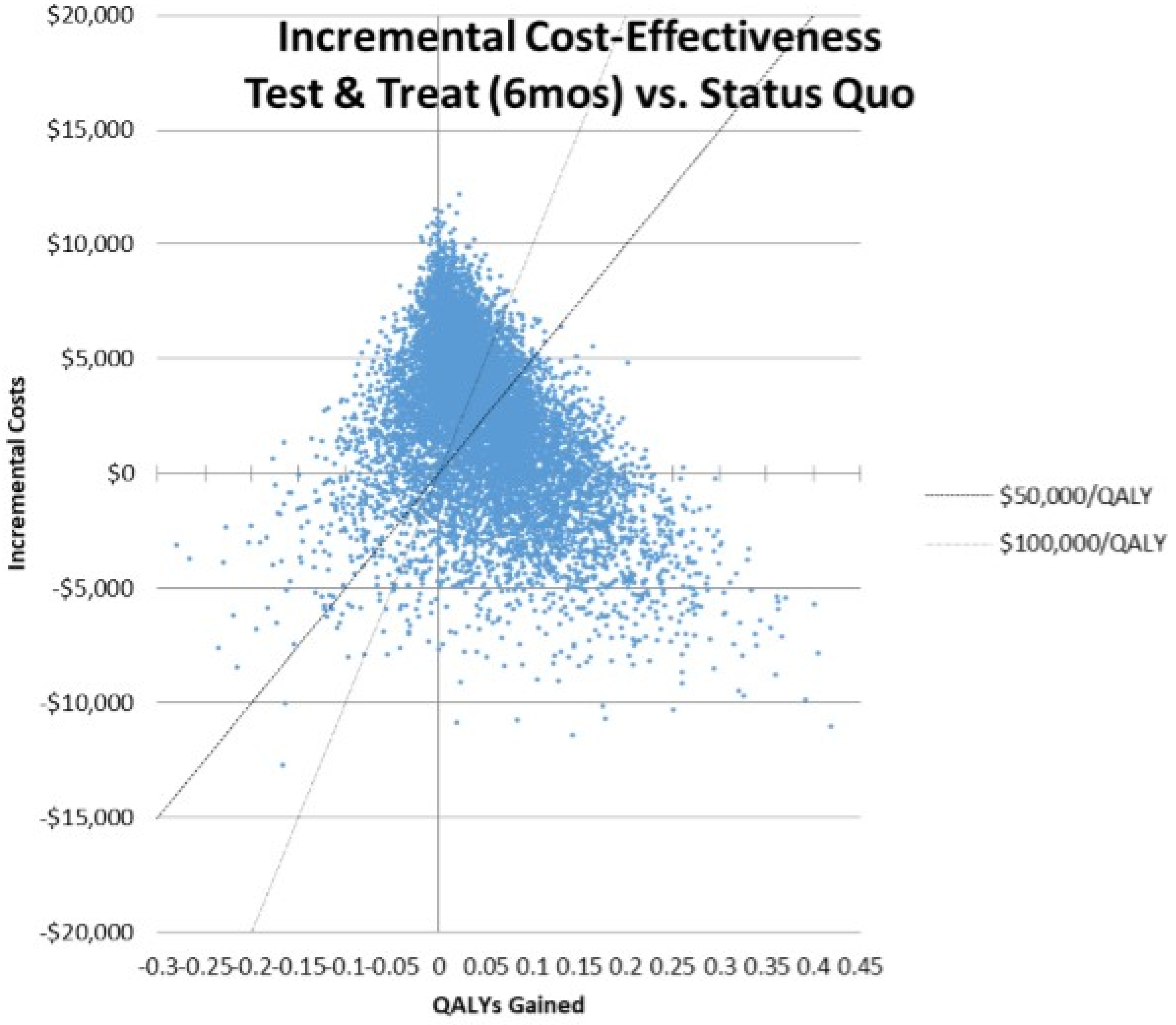

Supplement: Supplementary file 1 [file Image_1.pdf]
