# Supplementary material for: Cost-Effectiveness of MRI-Based Identification of Presymptomatic Autism in a High-Risk Population
Source: Front Psychiatry. 2020 Feb 19;11:60. doi: 10.3389/fpsyt.2020.00060 (PMC7042195; doi:10.3389/fpsyt.2020.00060)
Supplement: Supplementary file 2 [file Table_1.docx]

| **IMPACT SUMMARY** | | | | | |
| --- | --- | --- | --- | --- | --- |
| **Cost Category** | **2018 dollars** | | **Perspective** | | |
|  |  |  | **Societal** | **Healthcare** | **Education** |
| **Early Intervention** | | | | | |
| education | $106,227 | | **🗸** |  | **🗸** |
| productivity loss | $42,140 | | **🗸** |  |  |
| subtotal | | | **$148,367** | **$0** | **$106,227** |
| **Identification** | | | | | |
| MRI | $1,814 | | **🗸** | **🗸** |  |
| subtotal | | | **$1,814** | **$1,814** | **$0** |
| **Annual Costs (ages 6-17)** | **IQ < 70** | **IQ ≥ 70** |  |  |  |
| accommodation | $10,711 | $5,355 | **🗸** |  |  |
| education | $31,471 | $15,735 | **🗸** |  | **🗸** |
| medical services | $19,916 | $9,958 | **🗸** | **🗸** |  |
| non-medical services | $12,816 | $6,408 | **🗸** |  |  |
| productivity loss | $21,070 | $21,070 | **🗸** |  |  |
| subtotal (IQ < 70) | | | **$95,983** | **$19,916** | **$31,471** |
| subtotal (IQ ≥ 70) | | | **$58,526** | **$9,958** | **$15,735** |
| **Annual Costs (ages 18-21)** | | | | | |
| accommodation | $40,700 | $20,350 | **🗸** |  |  |
| education | $31,471 | $15,735 | **🗸** |  | **🗸** |
| medical services | $29,874 | $14,937 | **🗸** | **🗸** |  |
| non-medical services | $12,816 | $6,408 | **🗸** |  |  |
| employment support | $793 | $396 | **🗸** |  |  |
| productivity loss | $12,063 | $12,063 | **🗸** |  |  |
| subtotal (IQ < 70) | | | **$127,718** | **$29,874** | **$31,471** |
| subtotal (IQ ≥ 70) | | | **$69,889** | **$14,937** | **$15,735** |
| **Annual Costs (ages 22+)** | | | | | |
| accommodation | $40,700 | $20,350 | **🗸** |  |  |
| medical services | $29,874 | $14,937 | **🗸** | **🗸** |  |
| non-medical services | $12,816 | $6,408 | **🗸** |  |  |
| employment support | $793 | $396 | **🗸** |  |  |
| productivity loss | $12,063 | $12,063 | **🗸** |  |  |
| subtotal (IQ < 70) | | | **$96,247** | **$12,816** | **$29,874** |
| subtotal (IQ ≥ 70) | | | **$54,154** | **$6,408** | **$14,937** |
